# Presbyopia: addressing an urgent global need

**Published:** 2024-05-15

**Authors:** Pelin Munis, Jordan Kassalow, Mark Lorey

**Affiliations:** 1 Chief Executive Officer: RestoringVision, Miami, USA.; 2 Founder: VisionSpring and Co-founder: EYElliance, New York, USA.; 3 Vice President, Global Programs and Impact: RestoringVision, Washington, DC, USA.

**Addressing presbyopia – the most common global cause of visual impairment – can boost income and productivity while providing a gateway to wider eye health care.**

Presbyopia is the age-related deterioration of a person's near vision, making it difficult to focus clearly on reading materials or other nearby objects. It typically begins around age 40 due to a loss of elasticity of the lens of the eye. Near vision continues to deteriorate until about the age of 60 to 65, when the condition stabilises.

**Figure:**
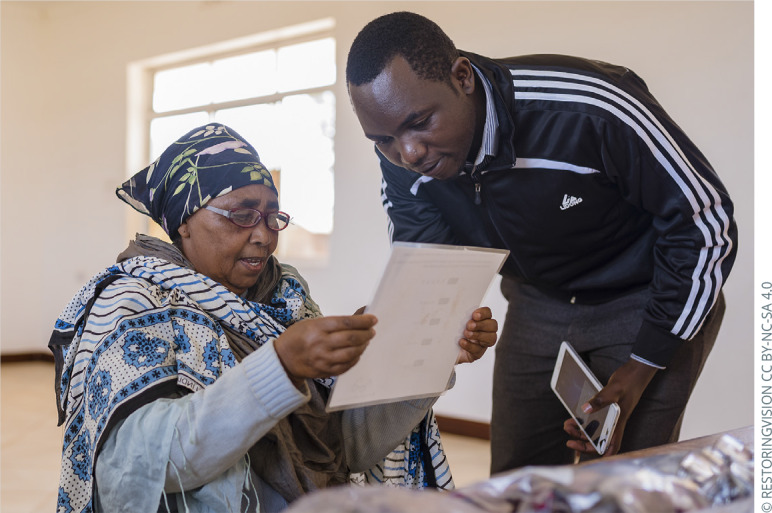
A woman is screened for presbyopia using a simple, printed one-page eye chart. tanzania

Presbyopia is the most common cause of vision impairment globally, impacting 1.8 billion people.^1^ Almost every person will experience presbyopia if they live into their 50s and beyond.

Presbyopia is addressed with the use of near-vision spectacles. These are commonly called ‘ready-made’ reading glasses, reading spectacles, or readers, although they have many uses in addition to reading. These spectacles are critical for people with presbyopia who rely on their ability to see close-up to work, read, learn, and perform day-to-day tasks and activities. People who also have problems with distance vision or astigmatism may need combined distance and near vision correction in the form of bifocal or progressive lenses, for example.

## The impact of unaddressed presbyopia

According to the World Health Organization's World Report on Vision^1^ (based on research by Fricke et al.^2^), more than 1 billion people have a vision impairment that could have been prevented or is yet to be addressed. Of these, more than 800 million people are estimated to be visually impaired due to uncorrected presbyopia and need only reading spectacles to be able to see clearly.^1^

It is estimated that 90% of people with uncorrected presbyopia live in low and middle-income countries. Without proper reading spectacles, they are being driven deeper into poverty as their blurred vision inhibits their ability to work, causing them to lose their jobs and livelihoods. Addressing presbyopia therefore presents a significant opportunity to improve income, productivity, and quality of life for hundreds of millions of people worldwide. Because it can be corrected at low cost with vision screenings and ready-made reading spectacles, presbyopia can and should be addressed on a much larger scale.

For people with presbyopia, receiving ready-made reading spectacles leads to positive changes in livelihoods, worker productivity, income, health, opportunities for education and lifelong learning, and overall well-being. In a randomised controlled trial (RCT), tea pickers who received reading spectacles were up to 32% more productive in their jobs than their peers in the control group.^3^ In another RCT, workers across a range of visually intensive jobs saw their incomes increase by nearly US $12 a month, an increase of 33.4%.^4^ In comparison, providing reading spectacles at scale can cost as little as US $2 per person, including programme costs (based on RestoringVision's 2023 programmes, which reached 4 million people in 106 countries); making it a highly cost-effective intervention.

“Almost every person will experience presbyopia if they live into their 50s and beyond.”

Many occupations require near vision, including tea and coffee pickers, textile workers, artisans, tailors, weavers, community health workers, midwives, mechanics, carpenters, plumbers, barbers, people who braid hair, and people who rely on their mobile phones for their livelihoods. In addition, in our work, many people have reported significant improvement in their quality of life when they are again able to read their holy scriptures and texts, communicate effectively through their mobile phones with family and friends, and engage in other activities that require near vision.

## A global issue

There is a growing understanding that uncorrected presbyopia and other forms of refractive error are a critical public health issue. Accordingly, World Health Organization (WHO) member states set an ambitious target to increase effective refractive error coverage by 40 percentage points by 2030. To accelerate the achievement of this target, WHO developed the SPECS 2030 initiative,^5^ which seeks to mobilise coordinated action across governments, civil society, and the private sector to increase spectacle coverage, especially in low and middle-income countries. The United Nations General Assembly also called for improving eye health and access to spectacles among its member states in a 2021 resolution, recognising the importance of eye health in achieving the Sustainable Development Goals.^6^

## What can be done?

Two key actions are needed:

1. Integrating presbyopia screening and correction at the community and primary care level
2. Aligning policies to support and accelerate the provision of spectacles for presbyopia

### 1. Integrating presbyopia screening and correction at the community and primary care level

WHO recommends that presbyopia correction is provided at the community and primary eye care level.^7^

In our experience, vision screening programmes to address presbyopia can readily be embedded within government and nongovernmental programmes and systems across a range of sectors, including health, education, agriculture, pensions, and social protection. This integration is a promising way to address presbyopia at scale, which can have an immediate and sustained impact in the lives of people reached and accelerate achieving the SPECS 2030 goal of increasing effective refractive error coverage. Due to the cost-effectiveness of programmes addressing presbyopia, these programmes can be implemented at scale. Several near vision testing methodologies can be used, including WHOeyes (bit.ly/WHOeyes), a new free mobile app by WHO, or a printed near vision eye chart with text, numbers, or symbols (like tumbling Es).

If a person's primary complaint is difficulty seeing at near distances (close-up), and the vision test confirms this, they can be given a pair of good quality, low-cost, ready-made reading spectacles to correct their vision impairment. If the individual presents with other eye conditions or concerns, they can be referred for a full eye examination.

**Figure:**
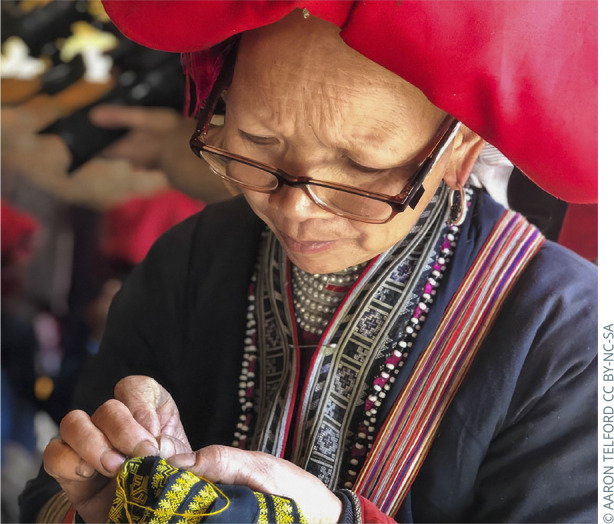
Access to reading spectacles can increase people's productivity and earnings. viet nam

Addressing presbyopia in this way can bring many people into the eye health care system for the first time. This provides a key opportunity to identify other critical eye conditions such as cataracts, glaucoma, or diabetic retinopathy, which are more prevalent in the age group of people typically affected by presbyopia.

Presbyopia programmes can also play an important role in building public awareness and understanding of wider eye health issues and risks. In our work, we have found that these programmes demonstrate the extent of visual impairment due to refractive error and the value of addressing this. In turn, this can lead to greater interest in the strengthening of comprehensive eye care programmes and greater allocation of resources for this purpose.

### 2. Aligning policies to accelerate the provision of spectacles for presbyopia

In many high-income countries, reading spectacles are sold ‘over the counter’ at low cost in shops and pharmacies, making them accessible and affordable to people who only need near vision correction. However, in many other parts of the world, including low and middle-income countries, reading spectacles are inaccessible and unaffordable for hundreds of millions of people, often due to policies that affect the cost of these spectacles, and how and where they can be provided.

There are two types of policies that can support addressing presbyopia on a larger scale.

1. **Policies on who can screen and dispense.** Many countries do not require reading spectacles to be dispensed only by trained eye health professionals. Instead, these spectacles can be provided by people with basic training in screening and dispensing or they can be made more widely available, so that individuals can select and purchase the pair that suits their visual needs.
2. **Policies on importing near vision spectacles.** Many countries allow the importation of reading spectacles duty-free or at a lower rate to ensure that these health products are accessible and affordable. This is an effective way to facilitate scaling up the response to presbyopia.

Unfortunately, a significant number of countries with very large populations of people with uncorrected presbyopia have not yet adopted these policies. It is recommended that these countries revise their policies in order to accelerate their achievement of the 40 percentage point increase in effective coverage for refractive error as outlined by WHO.^5^

**What about the need for a comprehensive eye examination?**

While there is consensus on the benefits of a comprehensive eye examination – every person should ideally have one every 1–3 years, depending on their age and health status – the number of optometrists and ophthalmologists in low- and/or middle-income countries is insufficient to meet the needs of the population. For example, fourteen low- and/or middle-income countries have fewer than one ophthalmologist per 1 million people, and another 31 countries have only 1–3 ophthalmologists per 1 million people. Ophthalmologists and optometrists also tend to be concentrated in urban areas.^8^

In these contexts, it is not feasible (or affordable) for everyone who needs a pair of reading spectacles to consult with an ophthalmologist or optometrist. Until the workforce can catch up with the need, it is vital for people to be screened and gain access to this essential product in a way that promotes overall eye health, as it can be the difference between someone sustaining their livelihood or not.

## References

[1] World report on vision. Geneva: World Health Organization; 2019. Available from: https://bit.ly/world-report-on-vision

[2] Fricke TR Tahhan N Resnikoff S Papas E Burnett A Ho SM Naduvilath T Naidoo KS. Global Prevalence of Presbyopia and Vision Impairment from Uncorrected Presbyopia: Systematic Review, Meta-analysis, and Modelling. Ophthalmol. 2018;125(10):1492–9. 10.1016/j.ophtha.2018.04.013 29753495

[3] Reddy PA Congdon N MacKenzie G Gogate P Wen Q Jan C Clarke M Kassalow J Gudwin E O’Neill C Jin L Tang J Bassett K Cherwek DH Ali R. Effect of providing near glasses on productivity among rural Indian tea workers with presbyopia (PROSPER): a randomised trial. Lancet Glob Health. 2018;6(9):e1019-e1027. 30049615 10.1016/S2214-109X(18)30329-2

[4] Sehrin F Jin L Naher K Das NC Chan VF Li DF. (2024) The effect on income of providing near vision correction to workers in Bangladesh: The THRIVE (Tradespeople and Hand-workers Rural Initiative for a Vision-enhanced Economy) randomized controlled trial. PLoS ONE 19(4): e0296115. 38568883 10.1371/journal.pone.0296115 PMC10990163

[5] Keel S Mueller A. WHO SPECS 2030 – A global initiative to strengthen refractive error care. Comm Eye Health J. 2024; Epub ahead of print. Available from: https://bit.ly/CEHJSPECS PMC11141117 38827975

[6] United Nations General Assembly, document A/75/L.108

[7] Package of eye care interventions. Geneva: World Health Organization; 2022.

[8] Resnikoff S Lansingh VC Washburn L Felch W Gauthier TM Taylor HR Eckert K Parke D Wiedemann P. Estimated number of ophthalmologists worldwide (International Council of Ophthalmology update): will we meet the needs? Br J Ophthalmol. 2020;104(4):588-592. 31266774 10.1136/bjophthalmol-2019-314336 PMC7147181

